# Crowdsourcing Opinions and Awareness of Upper Extremity Transplantation in the United States

**DOI:** 10.7759/cureus.60941

**Published:** 2024-05-23

**Authors:** Siam K Rezwan, Pathik Aravind, Joseph S Puthumana, Gerald Brandacher, Carisa M Cooney

**Affiliations:** 1 Department of Plastic and Reconstructive Surgery, Johns Hopkins University School of Medicine, Baltimore, USA

**Keywords:** vascularized composite allotransplantation, public awareness, service members, veterans, hand transplantation, upper extremity transplantation, vca

## Abstract

Introduction

As of 2008, the United States had 41,000 people living with upper extremity amputation. This number is projected to reach 300,000 by 2050. Human upper extremity transplantation (HUET) may become a more common treatment option with the potential to significantly improve the quality of life for certain amputees. Awareness and opinions regarding HUET among Americans, particularly in Veterans/Service Members (VSM) affiliates, are largely unknown.

Materials and methods

We administered a survey on Amazon Mechanical Turk (MTurk) workers. Eligible participants were US citizens aged ≥18 years; MTurk worker selection targeted workers who self-reported being a VSM. We used descriptive statistics to summarize study findings and Fisher’s exact and Wilcoxon's rank-sum tests for between-group comparisons.

Results

The survey was completed by 764 individuals, 604 (79.1%) of whom reported being aware of HUET. Among those familiar versus unfamiliar, a significantly higher proportion were aged ≤35 years (n=385, 64.0% vs. n=86, 53.7%; p=0.017), employed (n=523, 86.6% vs. n=114, 71.3%; p<0.001), and aware of their religion’s stance on organ/tissue donation (n=341, 54.5% vs. n=62, 38.8%; p<0.001). Amputees and/or respondents related to an amputee were more likely to be aware of HUET than individuals who were amputation naive (n=211, 90.6% vs. n=393, 74.0%, respectively; p<0.001), as were individuals with a personal or familial military affiliation (n=286, 85.4% with vs. n=318, 74.1% with no affiliation; p<0.001). The most reported HUET information sources were digital media (n=157, 31.2%) and internet (n=137, 27.2%).

Conclusions

Our survey of MTurk workers found greater awareness of HUET among individuals with a VSM or amputee connection. Our additional findings that the internet and academic sources, such as journals or reputable medical publications, were respondents’ preferred sources of HUET information emphasize the importance of vascularized composite allotransplantation (VCA) centers’ involvement in creating accurate and accessible content to help educate the public about this treatment.

## Introduction

Human hand and upper extremity transplantation (HUET) is the most frequently performed reconstructive procedure in the field of vascularized composite allotransplantation (VCA) to date [1]. VCA is a type of transplantation in which multiple tissue types, such as skin, nerves, muscle, and bone are transplanted as a single unit, usually from a brain-dead donor to a living recipient, and includes the face, upper and lower extremities, penis, and uterus, the last of which may also be procured from a living donor [2]. For individuals who have sustained devastating injuries, VCA represents a viable treatment option when conventional reconstruction is unable to restore normal form and function [3].

Since the first successful hand transplant was performed in 1998, more than 130 HUETs are known to have been performed worldwide [4]. With 41,000 upper limb amputees in the United States as of 2008 and a projected 300,000 living in the United States by 2050, HUET may become a more common treatment option with the potential to significantly improve the quality of life for certain amputees [5]. This may be particularly relevant to US Veterans and Service Members (VSMs) since the United States Armed Forces have been involved in armed conflicts for decades, putting service members at higher risk for trauma and amputations [6,7]. Despite numerous studies being published on the procedure and its outcomes, few studies assessing awareness and opinions of HUET in American civilians or VSMs have been published [8,9].

As HUET becomes more accessible to patients, understanding the public’s awareness of and opinions on this type of transplant is vital to best align upper extremity amputees with the reconstructive treatment that will best suit their lifestyle and needs [8]. We conducted the current cross-sectional study to characterize awareness and opinions of HUET in US residents/citizens with a particular interest in individuals who served or had a relative who served, in the United States Armed Forces. Domains of interest included respondents’ knowledge and awareness of HUET and sources of this knowledge, preferred sources to seek additional information on HUET, whether or not they or a close relative had an amputation, knowledge of other types of transplants, and sociodemographic characteristics that may be associated with awareness. Additionally, we explored any association these factors may have with respondents’ theoretical willingness to undergo HUET.

## Materials and methods

Starting in May 2020, we recruited participants using Amazon Mechanical Turk (MTurk), an online crowdsourcing marketplace used for research and data validation. As an increasingly popular tool to recruit survey respondents and yield high-quality data, general members of the public who registered on the MTurk platform (known as “MTurk workers”) selected and completed available tasks of interest, such as surveys, for payment [10]. The current literature has concluded that MTurk is a reliable tool comparable to conventional methods in conducting survey studies [11]. MTurk has been used previously to study research topics such as attitudes and beliefs relating to solid organ donation and transplantation in the United States [12].

We developed a 21-item study-specific survey, of which seven items pertained to participant sociodemographic factors, the survey was hosted on Qualtrics (Provo, UT: Qualtrics) and available via a hyperlink within MTurk (appendix 1). Two subject matter experts (GB, CMC) reviewed the survey before it was piloted to assess face validity and ensure respondents understood the survey questions as intended. The pilot group consisted of university faculty and students which was determined to be a reasonable pilot test group as MTurk workers are more likely to be college-educated and younger than 50 years compared to the general US population [13]. MTurk participant inclusion criteria were that respondents should be US residents aged ≥18 years. Prior to posting the survey, we selectively targeted MTurk workers who self-reported being VSMs through MTurk’s requester settings increased the promotion of the survey for this population. For the purposes of this study, VSMs and VSM affiliates were combined as family members often seek out information pertaining to care options for their service member relatives and may serve as caregivers and/or advocates for care, particularly for deployed service members or wounded VSMs [14,15].

Interested respondents first answered questions evaluating their awareness and knowledge of HUET, and identified their HUET information sources and resources from which they would want additional information on HUET. Respondents were then asked to name the types of human medical transplantation of which they were aware. Finally, respondents answered questions regarding their amputee status, knowledge of their religion’s stance on organ and tissue donation, and military affiliation followed by questions on their sociodemographic characteristics. This was followed by a brief description of the HUET procedure (appendix 2), an artist’s rendition of the procedure to enhance respondent understanding (appendix 1), and a final question regarding their theoretical willingness to undergo HUET. Respondents received $0.15 compensation upon survey completion.

Most questions evaluating respondent knowledge and awareness of HUET were binary (“yes/no”). The survey’s free response questions included, “If you knew about hand or arm transplantation, where/how did you learn about it?” and “Where would you want to find out more about hand or arm transplantation?” To analyze free-response questions, one author (SKR) developed a response classification scheme based on common response themes for each question. These classifications were then reviewed and approved by two co-authors (PA and CMC). All free responses were subsequently coded by one author (SKR) and reviewed by two co-authors (PA and CMC). Disagreement in response classification was resolved through discussion.

Descriptive statistics were used to summarize study data. Frequencies and percentages were calculated for categorical variables, and Fisher’s exact test was to compare categorical variables. Statistical significance was set at p<0.05. All statistical analyses were performed using Stata/SE 15.0 (College Station, TX: StataCorp). The study was acknowledged as exempt from review by the Johns Hopkins Medicine Institutional Review Board (#IRB00227912).

## Results

Between May 22 and June 6, 2020, 764 individuals completed the study survey. The majority of respondents were aged 18-35 years (n=471, 61.6%), female (n=401, 52.5%), white (n=533, 69.8%), and non-Hispanic (n=612, 80%); 30.5% (n=233) of respondents were missing a limb and/or were related to someone missing a limb and 43.9% (n=335) had a personal and/or familial military affiliation (appendix 3).

Among the 604 (79.1%) HUET-aware respondents, 570 (94.4%) knew its purpose, while 553 (97.0%) of those were specifically aware that it can be a treatment for upper limb absence or loss (appendix 4). When comparing respondents who were aware or had knowledge about HUET (“aware”) with those who did not (“unaware”), aware respondents were significantly more likely to be aged 18-25 years, employed, aware of their religion’s stance on organ and tissue donation, amputees or related to an amputee, a VSM themselves, or related to a VSM (appendix 3). Age stratification of respondents who indicated either being themselves or being related to an amputee, who indicated having been themselves or being related to VSM, or who indicated being aware of their religion’s stance on organ and tissue donation consistently displayed that younger respondents (aged 18-35 years) were more often aware of HUET (>60% of respondents) (Table 1).

**Table 1:** Awareness of HUET in three subgroups of respondents stratified by age. HUET: human upper extremity transplantation

Respondents with known amputee affiliation		
Aware of upper extremity transplants?		
Age (years)	Yes, n=211 (%)	No, n=22 (%)
18-25	42 (19.9)	3 (13.6)
26-35	92 (43.6)	9 (40.9)
36-45	40 (19.0)	3 (13.6)
>45	37 (17.5)	7 (31.8)
Respondents with known military affiliation		
Aware of upper extremity transplants?		
Age (years)	Yes, n=286 (%)	No, n=49 (%)
18-25	66 (23.1)	7 (14.3)
26-35	113 (39.5)	18 (36.7)
36-45	43 (15.0)	9 (18.4)
>45	64 (22.4)	15 (30.6)
Respondents aware of religion’s stance on organ and tissue donation		
Aware of upper extremity transplants?		
Age (years)	Yes, n=341 (%)	No, n=62 (%)
18-25	88 (25.8)	8 (12.9)
26-35	135 (39.6)	23 (37.1)
36-45	55 (16.1)	14 (22.6)
>45	63 (18.5)	17 (27.4)

**Figure 1:**
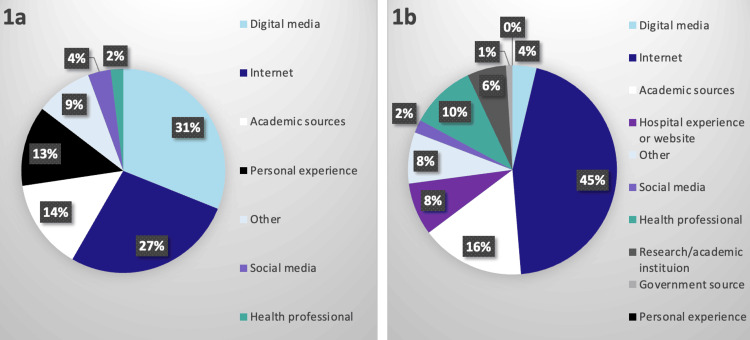
Sources from which respondents first learned of human upper extremity transplantation (a) and sources from which respondents preferred to learn more (b).

When asked about sources from which they learned about HUET, 503 (83.3%) respondents who provided valid data most frequently reported the source of TV/news/movies/documentaries (aka, digital media; n=157, 31.2%), internet (n=137, 27.2%), academic sources such as medical publications/journals (n=72, 14.3%), and personal experience such as learning about HUET from a friend, relative, or co-worker (n=64, 12.7%). Social media (n=18, 3.6%) and doctor/health care professionals (n=10, 2.0%) were the two least often reported sources. Regarding obtaining additional information, nearly half of the 592 participants who responded reported preferring the internet (n=265, 44.8%) followed by “reputable” or medical journals/publications (n=99, 16.7%), a doctor/health care professional (n=60, 10.1%), or a hospital website or experience (n=48, 8.1%) as desirable sources for additional information on HUET (Figures 1a, 1b).

Finally, after reviewing information on HUET’s advantages and disadvantages and an artist’s rendition of the HUET procedure, participants were asked about their hypothetical interest in receiving an upper extremity transplant. Of the 764 respondents, 305 (39.9%) were willing, 161 (21.1%) were not willing, and 298 (39.0%) were unsure and desired additional information. Demographic characteristics found to be significantly associated with willingness to undergo transplantation included Hispanic ethnicity (n=81, 53.3% vs. n=224, 36.6%), American Indian/Alaskan Native race (n=21, 87.5%), being aware of one’s religion’s stance on donation (n=192, 47.6% vs. n=113, 31.3%), having a bachelor’s degree or higher (n=215, 43.3%), being employed (n=268, 42.1% vs. n=37, 29.1%), amputee status (n=138, 59.2% vs. n=167, 31.5%) and military affiliation (n=168, 50.1% vs. n=137, 31.9%) (Table 2). Demographic characteristics negatively associated with willingness to undergo transplantation included African American or other races, being unaware of one’s religion’s stance on organ donation, and having a high school level of education or less (Table 2).

**Table 2:** Comparing factors if patients are willing to undergo UE transplant (yes vs. no). *Are you or any family member/relative an amputee or missing a limb? **Do you or any family member/relative have any past/present history of military affiliation? UE: upper extremity

Variables		Willingness to undergo UE transplant			p-Value
		Yes, n (%)	Maybe, n (%)	No, n (%)	
		305 (39.9)	298 (39.0)	161 (21.1)	
Demographic factors					
Age (years)	18-15	55 (32.7)	72 (42.9)	41 (24.4)	0.139
	26-35	135 (44.6)	98 (32.3)	70 (23.1)	
	36-45	63 (43.8)	58 (40.3)	23 (16.0)	
	>45	52 (34.9)	70 (47.0)	27 (18.1)	
Sex	Male	157 (44.0)	125 (35.0)	75 (21.0)	0.104
	Female	145 (36.2)	172 (42.9)	84 (20.9)	
	Non-binary	3 (50.0)	1 (16.7)	2 (33.3)	
Race	White	210 (39.4)	223 (41.8)	100 (18.8)	0.017
	Black/African American	29 (35.4)	29 (35.4)	24 (29.3)	
	American Indian/Alaskan Native	21 (87.5)	2 (8.3)	1 (4.2)	
	Asian	38 (39.6)	34 (35.4)	24 (25.0)	
	Native Hawaiian/Pacific Islander	0 (0)	2 (100)	0 (0)	
	Other	7 (25.9)	8 (29.6)	12 (44.4)	
Hispanic	Yes	81 (53.3)	47 (30.9)	24 (15.8)	<0.001
	No	224 (36.6)	251 (41.0)	137 (22.4)	
Socioeconomic factors					
Religion	None	59 (31.6)	84 (44.9)	44 (23.5)	0.667
	Christianity	213 (44.1)	178 (36.9)	92 (19.0)	
	Islam	11 (50.0)	4 (18.2)	7 (31.8)	
	Hinduism	5 (25.0)	11 (55.0)	4 (20.0)	
	Judaism	6 (40.0)	6 (40.0)	3 (20.0)	
	Buddhism	3 (30.0)	5 (50.0)	2 (20.0)	
	Other	8 (29.6)	10 (37.0)	9 (33.3)	
Awareness of religion’s position on organ and tissue donation	Yes	192 (47.6)	138 (34.2)	73 (18.1)	<0.001
	No	113 (31.3)	160 (44.3)	88 (24.4)	
Level of education	No formal education	1 (100)	0 (0)	0 (0)	0.002
	Lower than high school diploma	4 (33.3)	1 (8.3)	7 (58.4)	
	High school diploma	46 (32.6)	57 (40.4)	38 (27.0)	
	Associate degree	39 (34.2)	47 (41.2)	28 (24.6)	
	Bachelor’s degree or higher	215 (43.3)	193 (38.9)	88 (17.7)	
Employment status	Yes	268 (42.1)	243 (38.1)	126 (19.8)	0.016
	No	37 (29.1)	55 (43.3)	35 (27.6)	
Annual income	Less than $50,000	130 (35.8)	142 (39.1)	91 (25.1)	0.166
	$50,000-$99,999	131 (44.0)	117 (39.3)	50 (16.8)	
	$100,000-$149,999	31 (48.4)	21 (32.8)	12 (18.8)	
	≥$150,000	13 (33.3)	18 (46.2)	8 (20.5)	
Amputee/military affiliation status					
Amputee status*	Yes	138 (59.2)	68 (29.2)	27 (11.6)	<0.001
	No	167 (31.5)	230 (43.3)	134 (25.2)	
Military affiliation**	Yes	168 (50.1)	114 (34.0)	53 (15.8)	<0.001
	No	137 (31.9)	184 (42.9)	108 (25.2)	

For participants hypothetically willing to undergo HUET, the most common reasoning other than general interest and willingness (n=189, 24.7%) was the restoration of normalcy, functionality, and improved quality of life (n=97, 12.7%). Among those who were unwilling to undergo HUET, the primary reason was a desire for additional information (n=119, 15.6%) followed by fear of lifelong immunosuppressive treatment (n=38, 5.0%), preference of a prosthetic (n=23, 3.0%), fear of complications after the procedure (n=21, 2.8%), fear of the procedure itself (n=20, 2.6%), inadequacy of current research on HUET (n=12, 1.6%), and doubt regarding post-transplant function and effectiveness (n=3, 0.4%) (Table 3).

**Table 3:** Respondents’ reasons for being unwilling, willing, or unable to evaluate upper extremity transplantation after viewing the study-provided information on the procedure. QoL: quality of life

Opinion	n (%)
Not interested/negative response(s)	306 (40.1)
Undecided/want more/better information	119 (38.9)
Do not need/not in situation	70 (22.9)
Fear of lifelong challenges/treatment	38 (12.4)
Prefer prosthetic/generally not interested	23 (7.5)
Fear of complication/transplanted arm removal	21 (6.9)
Fear of procedure itself	20 (6.5)
Needs more research	12 (3.9)
Unsure of effectiveness or function post-transplant	3 (1.0)
Interested/positive response(s)	286 (37.4)
General interest/willingness/would consider	189 (66.1)
Regain normalcy/functionality/QoL	97 (33.9)
Not able to evaluate	172 (22.5)
Invalid	136 (79.1)
Other	19 (11.0)
Copy/paste	17 (9.9)

## Discussion

In this cross-sectional survey study intended to characterize trends in awareness and opinions regarding HUET in Americans, with particular interest in VSMs, we found that individuals who report being a VSM themselves or being related to a VSM are significantly (1) more aware of HUET (n=286, 85.4% vs. n=318, 74.1%, respectively) and (2) more likely to be willing to undergo HUET (n=168, 50.1% vs. n=137 31.9%, respectively) than respondents without a VSM affiliation. Similarly, individuals who have an amputation or have a relative with an amputation are more aware of HUET (n=211, 90.6% vs. n=393, 74.0%) and more willing to undergo transplant (n=138, 59.2% vs. n=167, 31.5%). Other characteristics associated with greater awareness and willingness to undergo HUET included younger age (≤35 years), higher level of education, being employed, and being aware of one’s religion’s stance on organ donation.

Given the US military’s involvement in armed conflicts over the past two decades and the Department of Defense’s interest in pursuing treatments that will restore wounded warriors to their pre-injury state [16], VSMs were a special population of interest in our study [6,7,17]. Indeed, our finding that self-identified VSMs are more aware of HUET may not be surprising [6,7]. Being deployed or having a loved one who is deployed may cause individuals to seek information regarding potential combat-related injuries and the reconstructive care available thereafter. Notably, the stories of several service members who lost two or more limbs and their recoveries, some of which included hand/arm transplantation, have been featured on television and the internet, two of the most common sources from which this study’s respondents stated learning about HUET [18,19]. Additionally, organizations such as the Wounded Warrior Project and Wounded Warrior Support Network connect injured service members and their families with each other and various resources [20]. Such networks may also help explain how, despite their relative rarity, several survey respondents reported knowing an individual who had undergone HUET.

Similarly, exposure to amputation and amputation care, through a personal history of amputation or a close relative with amputation, was associated with more awareness of and more willingness to undergo HUET. Several post-hand/upper extremity amputation care options are available including different types of prosthetics (e.g., body-powered, myoelectric, and cosmetic). However, previous studies have documented a lack of satisfaction with upper extremity prostheses that results in limited use or prosthesis abandonment [21,22]. This lack of satisfaction with current standard care options may lead to increased information-seeking behaviors by amputees and their relations/friends, thereby leading to their increased awareness of HUET and potential willingness to undergo this treatment. These differences help demonstrate the importance of conducting survey studies regarding HUET in appropriate patient (e.g., upper extremity amputee) populations.

Because of VCA’s generally intriguing nature, these transplants have been featured in print, internet, and television news stories; television talk shows; and other digital media. This and a 2009 finding that 61% of American adults seek out health information online may help explain why 58.4% (n=294) of respondents stated that they first heard of HUET through the combined sources of digital media and the internet with 44.8% (n=265) of respondents preferring to obtain more information via the internet. These results align with a 2020 study surveying US military veterans’ attitudes toward VCA transplantation wherein 44% of respondents reported media exposure to VCA within the past year; among these, 28% reported media exposure to hand/arm transplantation specifically [17]. As younger individuals tend to have increased exposure to online/social media sources, this may help explain our finding that younger respondents were more often aware of HUET [23]. However, the next most often preferred sources were reputable medical journals/publications followed by doctors or health care professionals, hospitals, and research institutions as these sources were generally recognized as being able to provide the most comprehensive and accurate information about HUET. VCA centers should be aware of these preferences as internet sourcing for HUET information outranks the areas of academic sources, hospital experiences, health professionals, research/academic institutions, and government resources combined. This emphasizes the importance of VCA center involvement in contributing to reliable, neutral, patient-centered online content, such as that created by Vanterpool et al. (2023, “Within Reach”) to help avoid misinformation about HUET [24,25].

In looking at sociodemographic factors, age, gender, religion, and annual income were not associated with respondents’ willingness to undergo HUET. This is consistent with a 2015 study that found individuals’ willingness to receive a kidney transplant was not associated with sociodemographic characteristics [26]. However, in addition to amputee status, sociodemographic factors we found associated with being willing to undergo HUET included race, ethnicity, awareness of one’s religion’s stance on organ donation, level of education, and being employed. MTurk workers who self-identified their race as American Indian/Alaskan Native or their ethnicity as Hispanic more frequently indicated that they would be willing to undergo HUET. Our finding that knowing one’s religion’s stance was associated with increased willingness to undergo transplantation reflects a 2011 review of the world’s religions that found no religious bans on hand transplantation [27]. Additionally, VSM affiliation was associated with increased willingness to undergo HUET - a finding confirmed by Ward et al. in 2020, who found most veterans “strongly support” HUET, and the majority of veterans were theoretically willing to receive an upper extremity transplant should they be affected by a severe disability or injury [17].

Finally, MTurk workers who had a bachelor’s degree or higher as well as those who indicated they were employed were significantly more likely to indicate a willingness to undergo HUET. Although there is very limited literature on willingness to undergo transplantation, this does relate to a solid organ transplantation study which showed that those willing to receive a kidney transplant had more education and higher rates of employment [28].

When asked for reasons for being willing/unwilling to theoretically undergo HUET, respondents who were willing stated a general willingness followed by a desire to regain normalcy, functionality, and quality of life; however, these points were included in our description of HUET as potential advantages. Similar to other studies [29], those who were unwilling stated desiring more and better information before deciding followed by fear of lifelong challenges and the treatment, preferring a prosthetic or being generally uninterested, or had concerns regarding complications or needing the transplant removed [23]. The majority of unwilling respondents desiring more information highlights the importance of providing effective and accurate HUET informational and educational materials to the public and relevant patient populations.

This study had several limitations. Although we piloted the survey tool to gather evidence of face validity, it was not validated further, and the pilot population of university students and faculty did not exactly represent the population of interest. While transplantation laterality is considered influential to individuals’ interest in HUET, we omitted this question from the survey and thus were unable to assess its role. As MTurk workers are provided with compensation upon survey completion, it is possible that respondents selected answer choices without answering honestly or expressing their true thoughts in order to complete the survey as quickly as possible. However, prior studies have found that MTurk workers comprise a reasonable potential study population and our analysis included screening out nonsensical or random entries [30]. Additionally, although MTurk workers have been shown to represent the US population relatively well, MTurk workers are more likely to be <50 years of age and have more education than the general US population [13]. This is reflected in this study population as the percentage of respondents with a bachelor’s degree or higher is almost twice that of the general US population. It is also possible that some MTurk workers had completed the survey twice due to the presence of some identical responses to free response questions; however, MTurk workers are assigned unique worker IDs that should have only allowed respondents to complete the survey once. Finally, because we included some educational information on HUET towards the survey’s end, it is possible that this information influenced respondents’ answers regarding questions, such as the types of transplantable organs with which they were familiar.

## Conclusions

In this study, which surveyed Amazon MTurk workers to assess their awareness of and theoretical willingness to undergo human upper extremity transplantation (HUET), we found that individuals who identified themselves as VSMs or were related to a VSM were more aware of and more likely to express willingness to undergo HUET compared to respondents without a VSM affiliation. Our findings were similar in individuals who have or are related to someone who has an amputation. Participants desiring additional information on HUET specified the internet and reputable journals/publications as their preferred sources. These data may help identify populations who are less aware of, less willing to undergo, or are in need of more information regarding HUET. In identifying these populations, surgeons, researchers, and VCA programs can develop accurate and accessible targeted educational tools to better educate patients about this treatment option.

## Figures and Tables

**Figure 2:**
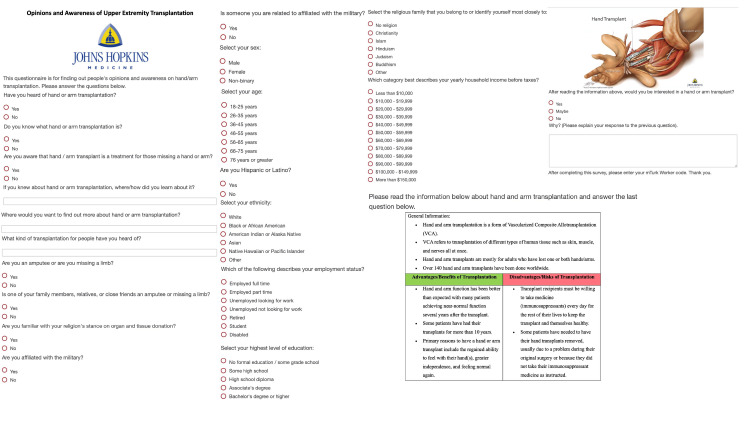
Study-specific survey hosted on Qualtrics and accessible via hyperlink on Amazon Mechanical Turk (MTurk). Image “Hand Transplant” embedded in survey copyrighted in 2012 by The Johns Hopkins Health System.

## Appendices

Appendix 1

Appendix 2

**Table 4:** Description of the upper extremity transplantation procedure provided to respondents.

General information: hand and arm transplantation is a form of vascularized composite allotransplantation (VCA). VCA refers to transplantation of different types of human tissue such as skin, muscle, and nerves all at once. Hand and arm transplants are mostly for adults who have lost one or both hands/arms. Over 140 hand and arm transplants have been done worldwide.	
Advantages/benefits of transplantation	Disadvantages/risks of transplantation
Hand and arm function has been better than expected with many patients achieving near-normal function several years after the transplant. Some patients have had their transplants for more than 10 years. Primary reasons to have a hand or arm transplant include the regained ability to feel with their hand(s), greater independence, and feeling normal again.	Transplant recipients must be willing to take medicine (immunosuppressants) every day for the rest of their lives to keep the transplant and themselves healthy. Some patients have needed to have their hand transplants removed, usually due to a problem during their original surgery or because they did not take their immunosuppressant medicine as instructed.

Appendix 3

**Table 5:** Respondent sociodemographic characteristics comparing those who were aware vs. unaware of human upper extremity transplant (HUET). *Are you or any family member/relative an amputee or missing a limb? **Do you or any family member/relative have any past/present history of military affiliation?

Factor		Total n=764 (%)	Aware of HUET n=604 (%)	Unaware of HUET n=160 (%)	p-Value
Demographic factors					
Age (years)	18-25	168 (21.9)	143 (23.9)	25 (15.6)	0.017
	26-35	303 (39.7)	242 (40.1)	61 (38.1)	
	36-45	144 (18.9)	114 (18.9)	30 (18.8)	
	>45	149 (19.5)	105 (17.4)	44 (27.5)	
Sex	Male	357 (46.7)	284 (47.0)	73 (45.6)	0.701
	Female	401 (52.5)	316 (52.3)	85 (53.1)	
	Non-binary	6 (0.8)	4 (0.7)	2 (1.3)	
Race	White	533 (69.8)	419 (69.4)	114 (71.3)	0.536
	Black/African American	82 (10.7)	69 (11.4)	13 (8.1)	
	American Indian/Alaskan Native	24 (3.1)	21 (3.5)	3 (1.9)	
	Asian	96 (12.6)	74 (12.3)	22 (13.8)	
	Native Hawaiian/Pacific Islander	2 (0.3)	2 (0.3)	0 (0.0)	
	Other	27 (3.5)	19 (3.2)	8 (5.0)	
Hispanic	Yes	152 (19.9)	131 (21.7)	21 (13.1)	0.014
	No	612 (80.1)	473 (78.3)	139 (86.9)	
Socioeconomic factors					
Religion	None	187 (24.5)	141 (23.3)	46 (28.8)	0.401
	Christianity	483 (63.2)	391 (64.7)	92 (57.5)	
	Islam	22 (2.9)	18 (3.0)	4 (2.5)	
	Hinduism	20 (2.6)	16 (2.7)	4 (2.5)	
	Judaism	15 (2.0)	11 (1.8)	4 (2.5)	
	Buddhism	10 (1.3)	9 (1.5)	1 (0.6)	
	Other	27 (3.5)	18 (3.0)	9 (5.6)	
Religion vs. donation aware	Yes	403 (52.7)	341 (54.5)	62 (38.8)	<0.001
	No	361 (47.2)	263 (43.5)	98 (61.2)	
Level of education	No formal education	1 (0.1)	1 (0.2)	0 (0.0)	0.732
	Lower than high school diploma	12 (1.6)	9 (1.5)	3 (1.9)	
	High school diploma	141 (18.5)	109 (18.1)	32 (20.0)	
	Associate degree	114 (14.9)	87 (14.4)	27 (16.9)	
	Bachelor’s degree or higher	496 (64.9)	398 (65.9)	98 (61.3)	
Employment status	Yes	637 (83.4)	523 (86.6)	114 (71.3)	<0.001
	No	127 (16.6)	81 (13.4)	46 (28.8)	
Annual income	Less than $50,000	363 (47.5)	281 (46.5)	82 (51.3)	0.227
	$50,000-$99,999	298 (39.0)	246 (40.7)	52 (32.5)	
	$100,000-$149,999	64 (8.4)	47 (7.8)	17 (10.6)	
	≥$150,000	39 (5.1)	30 (5.0)	9 (5.6)	
Amputee/military affiliation status					
Amputee status*	Yes	233 (100)	211 (90.6)	22 (9.4)	<0.001
	No	531 (100)	393 (74.0)	138 (26.0)	
Military affiliation**	Yes	335 (100)	286 (85.4)	49 (14.6)	<0.001
	No	429 (100)	318 (74.1)	111 (25.9)	

Appendix 4

**Table 6:** Respondents' awareness and familiarity with human upper extremity transplantation (HUET). *N=respondents who answered “yes” to question 1. **N=respondents who answered “yes” to question 2.

Variables	Total respondents	Yes (%)	No (%)
1. Have you heard of hand or arm transplantation?	764	604 (79.1)	160 (20.9)
2. Do you know what hand or arm transplantation is?*	604	570 (94.4)	34 (5.6)
3. Are you aware that hand/arm transplant is a treatment for those missing a hand or arm?**	570	553 (97.0)	17 (3.0)
